# We Can Dance If We Want To (with Safety Measures)

**DOI:** 10.1128/mbio.00295-22

**Published:** 2022-02-28

**Authors:** Susan Carson

**Affiliations:** a North Carolina State University, Raleigh, North Carolina, USA

**Keywords:** COVID-19, SARS-CoV-2, Lindy Hop, public health, social gatherings, viral transmission

## Abstract

This work considers the spread of SARS-CoV-2 during a multiday intensive dance camp occurring from 26 December 2021 to 1 January 2022 in Asheville, North Carolina. Approximately 370 dancers and performers were in attendance, and the data presented are the result of an anonymous survey distributed 10 days following the event. While some transmission occurred during the time span of the event, it appears that the majority of transmission occurred either through the result of individual interactions or activities outside the formal dance event rather than due to a buildup of airborne viral particles in the event space.

## EDITORIAL

Lindy Focus was a 6-day event focused primarily on Lindy Hop partner dancing. It took place in Asheville, North Carolina, from 26 December 2021 to 1 January 2022, a period when cases of Omicron, a highly transmissible, vaccine-resistant variant of SARS-COV2 was rising rapidly throughout the United States. Proof of vaccination and mask use were required in all of the dances, classes, and open practice sessions with a few exceptions to masking for performers. Approximately 370 attendees participated over the course of the week, which included registrants, volunteers, and performers. Not all attendees attended all days. In addition to organized dances and classes, most attendees roomed at the hotel associated with the event.

The information presented below is based on the self-reported results of an anonymous survey that was provided to all attendees 10 days following the event. Two hundred six individuals responded to the survey. Based on self-reported habits and personal observation, mask compliance was high during the organized dances, classes, and practice sessions. Although booster vaccines were not required for the event, 81.5% of survey respondents indicated that they received a booster prior to attendance (75% of the COVID-positive group and 81% of the COVID-negative group). In addition, 71.8% of respondents indicated they took COVID tests whether they had symptoms or not; 25.7% of respondents had no symptoms and did not test. While some level of asymptomatic transmission may have been undetected, this level of testing may capture more cases than reports of cases in the general public (1; and as referenced by the Centers for Disease Control and Prevention, https://www.cdc.gov/coronavirus/2019-ncov/cases-updates/burden.html).

Twenty people (9.7% of respondents) reported a positive COVID test between 26 December 2021 and 10 January 2022, excluding two people who responded that they tested positive in the 7- to 10-day range after the event and have reason to believe they were exposed after the event. Two individuals who tested positive during the event later learned their exposure had occurred prior to the event. If we exclude these two individuals, 8.7% of respondents (18 people) believe they contracted SARS-CoV-2 in the time frame of the event.

There are several factors to consider in thinking about the overall rate of transmission and whether transmission was more likely to occur during the dances or through interactions that took place outside the organized event. The survey asked attendees about possible risk behaviors, both inside and outside the organized event. Sixty-one percent of all respondents spent time indoors without a mask in public areas (e.g., hotel bars, restaurants, etc.); this number was similar among those who tested positive for COVID and those who did not. Fifty percent of COVID-positive individuals reported at least one close contact (individuals who the respondent interacted with in the absence of masks) who was also infected. All individuals who tested positive had at least one identified risk factor beyond the official Lindy Focus activities.

There is one piece of evidence, other than the reporting of additional risk factors, that leads me to believe that while some transmission may have occurred during the formal dance events, it is more likely to have occurred as a result of individual interactions rather than a buildup of airborne viral particles. Performers who needed to be unmasked during the large main dances were asked to identify themselves. This includes musicians who had to be unmasked for hours each night in the main dance and dancers who performed for a shorter period between band sets. Sixteen individuals responded that they performed without masks; one of these is excluded because they indicated they performed in the associated event that served snacks, not the main dance. Of the 15 remaining performers, only 1 tested positive for COVID (6.6%); this individual also reported additional risk factors. Had the primary cause of transmission been buildup of airborne viral particles, we would expect the transmission to unmasked individuals to be greater than to masked individuals.

Mask type may have played a role in transmission that potentially occurred during interactions in the organized event. Cloth masks are known to provide inferior protection than N95-, KN95-, and KF94-style masks (2, 3), and surgical masks offer reduced protection if they are not adjusted to fit closely to the face (4; and as recommended by the Centers for Disease Control and Prevention, https://www.cdc.gov/coronavirus/2019-ncov/prevent-getting-sick/types-of-masks.html). The use of cloth masks was higher in the infected group than the not-infected group (40% compared to 28%), and the use of surgical masks was also higher in the infected group than the not-infected group (20% compared to 15%).

Based on the information described, I offer several recommendations for organizers and individuals to minimize the risk of SARS-CoV-2 transmission for both stand-alone social dances and multiday dance events.

Organizers should (i) require the use of high-quality masks that fit closely to the face, such as N95, KN95, or KF94 masks. The fit of surgical masks should be adjusted using an approved method such as the knot and tuck or wearing a tightly fitted cloth mask over it. Cloth masks and masks with exhale valves should not be permitted; and (ii) not plan any activities where masks would be removed. If an event provides food indoors, it is not a fully masked event.

Individuals should be aware that event organizers only have loose control over a large crowd, regardless of what rules are in place. Additional actions attendees can take to reduce risk are to (i) wear the most protective mask possible; (ii) accept dances only with others who are wearing high-quality, well-fitted masks; (iii) keep your mask on indoors, partaking in snacks and drinks outdoors; (iv) for multiday events, limit the number of close contacts with whom you interact without a mask and avoid being unmasked in indoor public spaces; and (v) have a plan for reducing possible transmission to others following the event by consistent mask use in indoor public spaces, postponing visits with vulnerable populations, and scheduling a PCR test for 3 to 5 days after the event.

For many who participate in social dance, it is a primary form of social interaction and exercise, so its absence has potential to impact both mental and physical health. The media have covered a number of social events that led to a high degree of SARS-CoV-2 transmission (5, 6; and as reported by the Norwegian Institute of Public Health, https://www.fhi.no/en/news/2021/preliminary-findings-from-outbreak-investigation-after-christmas-party-in-o/). However, those cases did not require both vaccination and consistent mask wearing; thus, dancers did not have information available to weigh their risks. Based on the evidence gathered through this survey, I hope organizers and individuals will be better equipped to weigh risks and have increased awareness about strategies to reduce risk.[Fig fig1]

**FIG 1 fig1:**
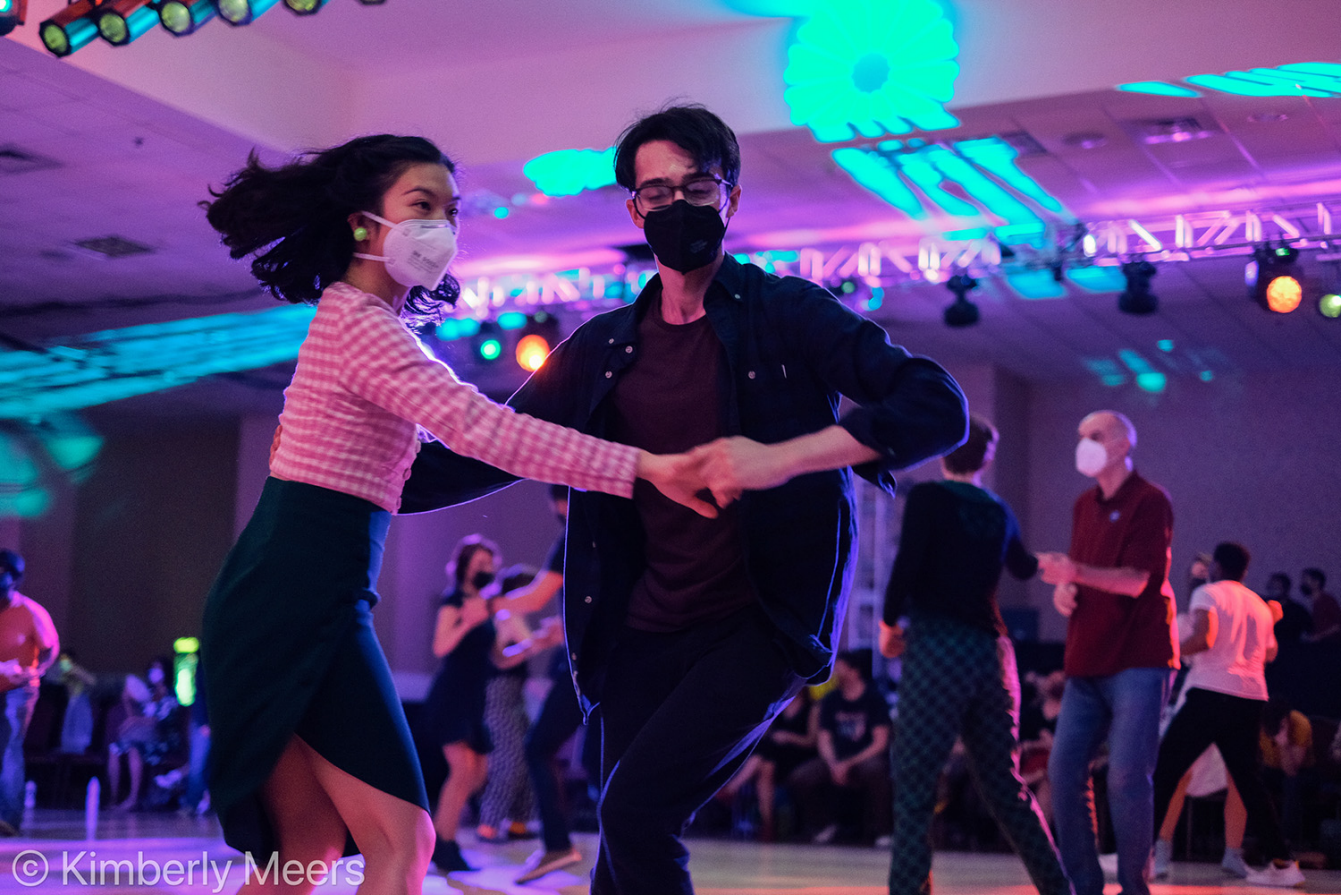
Lindy Focus XIX, December 28, 2021, Asheville, NC. Photograph courtesy of Kimberly Meers.
